# Euro Conditionality Hinges on Positive Convergence

**DOI:** 10.1007/s10272-022-1081-2

**Published:** 2022-10-10

**Authors:** Michala Marcussen

**Affiliations:** Societe Generale, 17 Cours Valmy, 92972 Paris La Défense, France

**Keywords:** F36, H63, H68

## Abstract

The present cocktail of a bleak economic outlook combined with high inflation and rising interest rates has raised questions as to whether a new euro debt crisis might emerge. Euro area sovereign spreads have widened, but not to “unwarranted” levels, unlike the situation that prevailed during the European debt crisis. A replay of the funding fears, which drove the euro area debt crisis a decade ago, seems unlikely today with the extensive toolkit now in place.

Once the euro area debt crisis faded, so too did the sense of urgency to complete the banking union and deepen the capital markets union, commonly agreed necessary to foster positive convergence. This is a concern as the cornerstone of conditionality, which underpins the toolkit keeping euro area spreads in check, ultimately depends on economic growth. The present energy and climate crises offer an opportunity to put positive euro convergence back on track; however, this requires not only a favourable outcome from the ongoing overhaul of the EU fiscal framework, but also the completion of Europe's financial architecture to secure a sufficient flow of private capital.

## "Unwarranted" spread widening unlikely

Euro area spreads have been widening again since late 2021, with Italy centre stage in the market debate, not only due to concerns about high general government debt levels, but also due to the sheer size of the Italian bond market, ranking among the largest in the world.

Top of the list of factors driving this spread widening is a weak economic growth outlook, combined with higher inflation expectations. This configuration is observed across the euro area, leaving the European Central Bank (ECB) with the difficult task of taming inflation, without plunging the euro area into a painful recession. This challenge is not unique to the euro area but observed across several of the major advanced economies, and not least the United States. Other major advanced central banks, however, do not face the same challenge as the ECB in leaning against "unwarranted" sovereign spread widening.

In contrast to the debt crisis of a decade ago, the euro area today has a far more effective toolkit at hand, with the establishment of the European Stability Mechanism (ESM) in 2012, the banking union (set in motion in 2012, albeit still incomplete) and several new ECB instruments. Taking quick stock of the ECB instruments, back in June 2012, then ECB President Mario Draghi promised to do "whatever it takes" to preserve the integrity of the euro, and soon afterwards the ECB delivered Outright Monetary Transactions (OMT), backed by the strict conditionality of an appropriate European Stability Mechanism (ESM) programme for member states in need of eventual support. The ECB went on to demonstrate that it could also conduct quantitative easing, with large scale purchases of government bonds, just like its global peers, to ensure sufficiently accommodative monetary policy.

When the COVID-19 pandemic struck in early 2020, the ECB introduced the Pandemic Emergency Purchase Programme (PEPP), which allowed purchases to be "conducted in a flexible manner on the basis of market conditions and with a view to preventing a tightening of financing conditions that is inconsistent with countering the downward impact of the pandemic on the projected path of inflation" (ECB, n.d.). Net asset purchases were discontinued in March 2022, but the ECB plans flexible reinvestment of securities maturing under PEPP at least until the end of 2024 (ECB, 2022b). On 21 July 2022, the ECB further strengthened its arsenal with the new Transmission Protection Instrument (TPI), backed by the conditionality of a cumulative list of criteria, including compliance with the EU fiscal framework (ECB, 2022c). Like the OMT, TPI purchases are not restricted *ex ante*, and the Eurosystem accepts the same (*pari passu*) treatment

* The views expressed are attributable only to the author and to not any institution with which she is affiliated.

**Michala Marcussen**, Société Générale, Paris, France.

as other creditors. Both programmes, moreover, allow for such purchases to be sterilised.

TPI purchases, however, have the additional flexibility of being able to purchase public sector securities with a remaining maturity of one to ten years, compared to one to three years for the OMT, and do not require the conditionality of an ESM programme. Importantly, the ESM Treaty notes that "In accordance with IMF practice, in exceptional cases an adequate and proportionate form of private sector involvement shall be considered in cases where stability support is provided accompanied by conditionality in the form of a macro-economic adjustment programme" (ESM, 2012). The TPI makes no mention of private sector involvement.

As recently observed by Edward Scicluna, Governor of the Central Bank of Malta, in commenting the TPI in Eurofi, "While there are clear monetary policy justifications for this instrument, drawing the line between warranted and unwarranted interventions represents a major challenge" (Scicluna, 2022). This author can only agree, but a few observations can nonetheless be made.

As a simple proxy for "unwarranted" spread widening, we turn to BBB corporate bond spreads, with the idea of capturing the "warranted" spread widening linked to an overall deterioration of economic conditions and related increase of credit risks (see Figure 1). The metric, however, does have imperfections. Arguably, an unwarranted deterioration of sovereign spreads in the euro area could, via doom-loop mechanisms, also spill over to corporate spreads. A comparison to the US BBB corporate bond spread should largely exclude the effect of a weaker euro area sovereign weighing on its domestic economy and hence also on corporate issuers. The US economy could, however, be at a different point in the economic cycle than the euro area, and thus fail to correctly capture the broader deterioration of credit risk, driving a "warranted" spread widening in the euro area.

At the current juncture, both the US and euro area face a weak economic outlook and monetary policy tightening, and neither of these simple metrics suggest any evidence of "unwarranted" widening of Italian sovereign spreads, i.e. not explained by the overall deterioration of credit.

## A challenge for the ECB's balance sheet

"Unwarranted" spread widening, however, is not just a challenge for sovereigns but also for the broader lending channels to the real euro area economy. A quick glance at swap spreads shows that these have widened significantly and even to levels above those observed during the euro area debt crisis. The present swap spread widening is more likely a reflection, in particular, of collateral shortages and hedging demand. While unlikely to be the cause of any new debt crisis, the current market frictions, notably in money markets, raise questions on the ECB balance sheet management and may push the ECB to consider new tools.

Figure 1
**Euro area: Ten-year sovereign spreads and BBB corporate spreads**
Basis points

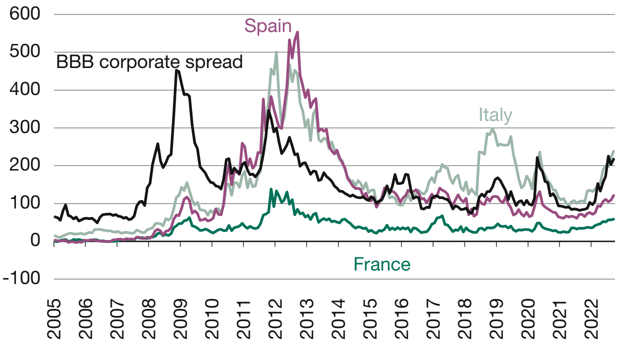

Source: Refinitiv, SG Economic and Sector Studies.

Euro area banks are in far stronger financial shape today, as also reflected in credit default swap (CDS) spreads (Figures 2 and 3). Mirroring the general pricing of credit risks, these have recently widened but remain well below levels observed during the euro area debt crisis. Furthermore, we note that the structural push towards central clearing, where counterparties post collateral, should – all else being equal – help reduce counterparty risk.

A more likely explanation for the present widening of euro area swap spreads, in excess of what has been observed in the US, is linked to various technical factors and structural differences in the respective financial systems. The euro area is notably more dependent on bank lending, and the fragmentated structure of collateral in the euro area is due, not least, to the absence of a single safe asset comparable to US Treasuries.

It is interesting, with this backdrop in mind, to consider the key role that the Eurosystem balance sheet today plays for funding and collateral transformation, and its key role in the toolkit.

Back in December 2011, the ECB announced the first non-standard LTRO with a maturity of 36 months and the option of early repayment after one year, along with the easing of collateral rules. This formula has since been

Figure 2
**Banks five-year credit default swap**
Basis points

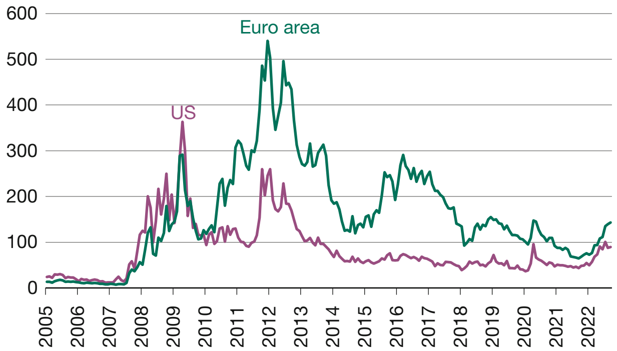

Source: Refinitiv, SG Economic and Sector Studies.

Figure 3
**Two-year swap spreads**

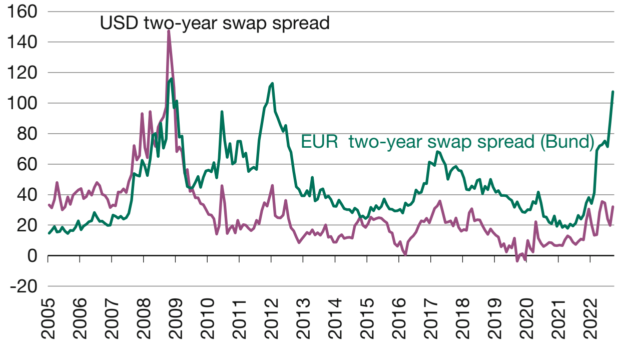

Source: Refinitiv, SG Economic and Sector Studies.

used numerous times, with characteristics adopted to the different situations. The tool was also drawn upon in response to the COVID-19 pandemic and LTROs presently stand at €2,215bn out of €8,759bn total assets on the consolidated ECB balance sheet. This compares to securities held for monetary policy purposes of €4,955bn (ECB, 2022a).

A significant chunk of the financing for these operations comes from reserves, which presently stand at €4,803bn. Euro area banks, in turn, hold a sizable amount of the excess reserves[1] as the High-Quality Liquid Assets (HQLA) required to meet financial regulations. To the extent that the ECB accepts non-HQLA collateral, the Eurosystem not only plays a role for longer-term bank funding via the supply of LTROs, but also for collateral transformation (Grandia et al., 2019). This observation on collateral transformation holds true also for the Eurosystem's asset purchases. Excess reserves are available only to banks and not the broader financial system, and Eurosystem asset purchases are regularly blamed for the shortage of supply of the euro area's safest sovereign asset, namely German government issues. This argument should be complemented by the related increase of reserves, the safest assets, albeit only accessible to the banking system.

A related concern is that the ECB will struggle to transmit its monetary policy to the money markets. The latest rate hike did not feed fully through to the euro short-term rate (€STR), the euro benchmark for bank's unsecured overnight borrowing costs, but overall the result was satisfactory. No doubt the ECB's decision of 8 September 2022 to temporarily (until 30 April 2023) remove the 0% interest rate ceiling for remuneration of government deposits at the Eurosystem was helpful in this respect.

A recent ECB working paper (Eisenschmidt et al., 2022) suggested that allowing over-the-counter customers access to inter-dealer repo platforms and/or allowing nonbanks access to a secured deposit facility, similar to the Fed's overnight reverse repo agreement, may be an effective way to improve monetary policy transmission. Traditionally, the ECB has stayed away from such measures for fear of disintermediating banks, and not least in a still fragmented system that is heavily dependent on bank lending. A further question if the ECB were to offer such an instrument, is what collateral would it supply for reverse repo operations.

The ECB has announced that it plans to continue asset purchase programme reinvestment for now and PEPP reinvestment at least until the end of 2024. Moreover, the Governing Council has stated that it continues to monitor bank funding conditions, a hint that an extension of LTROs could be announced if deemed necessary.

While the points raised above are reassuring in as much as there is little indication of any systemic financial stress, important questions still loom as to how the ECB will manage its large balance sheet in a period of monetary policy tightening. As noted by ECB Board Member Panetta in his speech on 25 May 2022,

> although we have plenty of experience of how asset purchases and policy rates can reinforce each other as part of an easing strategy, we have no experience of the reverse scenario in the euro area. And the experience of other major central banks, limited as it is, is

1 Reserves in excess of the minimum reserve requirements.

unlikely to be transferable to the euro area given the unique nature of our economic, financial and institutional set-up (Panetta, 2022).

## Conditionality is the cornerstone

With the euro area now in full possession of an effective toolkit to stem funding risks as long as conditionality is respected, the willingness to respect that conditionality becomes the cornerstone in preventing a new euro area debt crisis. It is no wonder, in this context, that national political debates are now scrutinised by markets for hints of rhetoric that may challenge the respect of conditionality.

The Eurobarometer is a popular source of information for those seeking to gauge these trends, with the advantage that these indicators have a long history, but with the drawback of being updated only twice a year, leaving markets to make their own assessment, drawing on rhetoric, polling and economic trends to fill the gap.

Just zooming in on Italy, the latest Eurobarometer from the summer of 2022 shows support for the single currency at 71%, just below the EU27 average of 72%. Mirroring this positive trend, we also note that many of the political parties previously calling for euro exits are no longer doing so. Overall attitudes towards the EU, however, remain morose with 46% seeing the EU conjuring up a positive image, 38% neutral and 16% negative.

Several studies have sought to understand the drivers behind such trends, and the following list sets out some of the most popular explanations: (1) the overall state of the economy, (2) perceptions of European solidarity in dealing with problems, (3) the perceived risk of the euro being a source of inflation and instability and (4) eurosceptic positions expressed by political leaders. As noted above, this latter factor comes with a certain level of circularity as the rhetoric of politicians may also be influenced by their own perceptions of public opinion.

Just looking at some of the recent trends, and still zooming in on Italy, on growth dynamics, we observe that the decline in Italian GDP per capita relative to that of Germany on a purchasing power parity basis is no longer in freefall. Moreover, setting aside the pandemic related recession in 2020, overall real GDP growth outcomes have also improved, and unemployment rates have declined significantly.

The EU's handling of the pandemic, and not least the introduction of the Next Generation EU facility worth up to €750 billion (in 2018 prices), likely offered further reas-

Figure 4
**Italy: Key economic indicators**

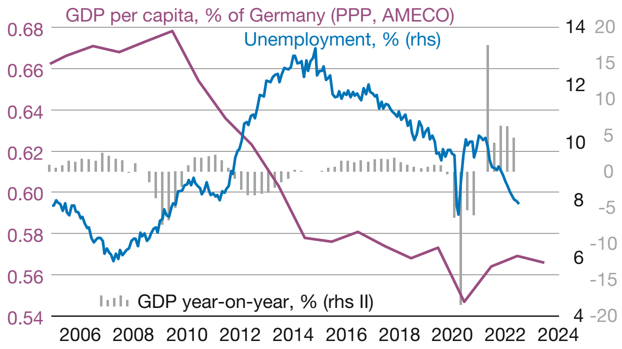

Source: Refinitiv, SG Economic and Sector Studies.

surance on European solidarity and thus the merits of the EU. ECB measures were likely a further positive, and not least with the PEPP and LTROs.

On inflation, the recent surge is more likely blamed on Russia and soaring energy costs than on Europe. The question of how the EU tackles the issue of electricity prices and, more broadly, manages solidarity in energy supply over the winter could become a new test for public attitudes towards the EU, and not just in Italy. The ECB, moreover, faces a challenge in tightening monetary policy without triggering a recession, particularly a recession with asymmetrical effects across member states.

Baccaro et al. (2021) further suggested that opposition to austerity in Italy outweighs support for the euro. This research was conducted at a time when conditionality came with an ESM programme and thus with direct external oversight. The conditionality attached to the ECB's new TPI instrument, does not involve the same force of direct oversight but does require that national governments respect the overall EU fiscal framework. Understanding public attitudes towards this type of conditionality will require more work that is probably still premature to conduct, not least given the ongoing review of the EU fiscal framework.

There is little disagreement that the EU's current fiscal framework needs reform to reach better fiscal outcomes and to secure better management of the inevitable trade-offs between limiting fiscal risks and stabilising economic output. Agreeing upon such reform will no doubt be politically challenging but has become all the more important with the new TPI now conditioned on the respect hereof and with the enforcement of the fiscal framework suspended under the so-called "general escape clause" of the Stability and Growth Pact, until the end of 2023.

The Commission has nonetheless called for a prudent approach, favouring investment over current expenditures.

Numerous proposals have been set forth as to how the EU fiscal framework may be improved, and one of the most interesting questions is whether the new framework will contain a permanent central fiscal tool, inspired by the NGEU, and as called for in a recent IMF paper (Arnold et al., 2022). Such a tool could help support the urgently needed investment, not least in developing a new green energy infrastructure. The hope is, moreover, that Germany with its urgent need to revamp its unsustainable economic model, and overly dependent on cheap fossil fuels, may today be more open to such suggestions than in the past. Indeed, the observations made above on public attitudes towards the EU likely also hold true for Germany. It is key for the euro area to secure "positive convergence", i.e. where member states enjoy positive per capita growth and converging overtime to the higher levels across member states.

A further interesting question in respect to the new EU fiscal framework is also the role that the National Energy and Climate Plans (NECPs) may take on. The Commission currently monitors these and reports on progress every two years as part of the State of the Energy Union Reports. The next set of NECPs are due in 2023 and will be key for investor visibility.

Welcome as a permanent NGEU-like feature would be, it is unlikely that it would be large enough to cover the significant investment needs. The EU Commission estimates investments needs of €520 billion per annum out to 2030 (€390 billion for decarbonisation and €130 billion for other environment goals) and a further €125 billion per annum for the digital transition. Further significant investment is also needed for climate adaptation, necessary to deal with the damage already done to the climate system and the increased occurrence of extreme weather.

The European financial system thus needs to be in a position to supply such substantial amounts of new net private financing, and this requires completion of a banking union and a deep capital markets union. It is unlikely, moreover, that a further increase of the ECB balance sheet would be able to fill this gap. Indeed, developing green technologies, ensuring energy security and growing new green businesses will require green capital and not just green bonds and green bank loans. An ECB Working Paper found that $CO_2$ emissions per capita are lower in economies that are more equity funded (De Haas and Popov, 2019). Europe needs to reduce its debt-equity bias.

The winning paper of the ECB's 2022 Young Economist Prize states:

> Using a macro model consistent with micro data on European firms' external financing over their lifetime, I find that financial frictions lead to big output losses, due mainly to young firms' early exits. Middle-income countries see 60% higher losses than high-income countries (Kochen, 2022).

This further illustrates the need for the right type of funding to grow new green businesses.

The ECB, for its part, can support green EU policies with its new framework for the greening of monetary policy. For now, the focus in terms of monetary policy instruments is on introducing climate criteria on private asset purchases and private collateral provided, but it does raise an interesting question as to whether such criteria should also be introduced on public paper, adding a new dimension to the ECB conditionality.

For European policymakers preparing and deciding on these new designs, keeping in mind what drives public attitudes to conditionality is key. Designs that ensure positive convergence and good solidarity will keep future crises at bay. Albeit at times challenging and rarely linear in progress, this is the essence that underpins the long history of European integration.

## References

[CR1] Arnold, N. G., R. Balakrishnan, B. B. Barkbu, H. R. Davoodi, A. Lagerborg, W. R. Lam, P. A. Medas, J. Otten, L. Rabier, C. Roehler, A. Shahmorad, M. Spector, S. Weber and J. Zettelmeyer (2022), Refroming the EU Fiscal Framework: Strengthening the Fiscal Rules and Institutions, *IMF Departmental Paper*, DP/2022/014.

[CR2] Barro L, Bremen B, Niemans E (2021). Till austerity do us part? A survey experiment on support for the euro in Italy. Sage Journals, European Union Politics.

[CR3] De Haas, R. and A. Popov (2019), Finance and carbon emissions, *ECB Working Paper Series*, 2318.

[CR4] Eisenschmidt, J., Y. M and A. L. Zhang (2022), Monetary policy transmission in segmented markets, *Working Paper Series*, 2706.

[CR5] European Central Bank (n.d.), Pandemic emergency purchase programme (PEPP), https://www.ecb.europa.eu/mopo/implement/pepp/html/index.en.html (21 September 2022).

[CR6] European Central Bank (2022a, 13 September), Consolidated financial statement of the Eurosystem as at 9 September 2022, Press release.

[CR7] European Central Bank (2022b, 8 September), Monetary Policy Decisions, Press release.

[CR8] European Central Bank (2022c, 21 July), The Transmission Protection Instrument, Press Release.

[CR9] European Stability Mechanism (2012), Treaty Establishing the European Stability Mechanism, Consolidate Version.

[CR10] Grandia, R., P. Hänling, M. Lo Russo and P. Åberg (eds.) (2019), Availability of high-quality liquid assets and monetary policy operations: an analysis for the euro area, *ECB Occasional Paper Series*, 218.

[CR11] Kochen, F. (2022), Finance Over the Life Cycle of Firms, https://www.ecb.europa.eu/pub/conferences/ecbforum/shared/pdf/2022/kochen_paper.en.pdf (21 September 2022).

[CR12] Panetta, F. (2022, 25 May), Normalising monetary policy in non-normal times, Speech at a policy lecture hosted by the SAFE Policy Center at Goethe University and the Centre for Economic Policy Research.

[CR13] Scicluna, E. (2022), Steering between Scylla and Charybdis once again, *Views The Eurofi Magazine*, September 2022.

